# Anatomical and Pathological Observation and Analysis of SARS and COVID-19: Microthrombosis Is the Main Cause of Death

**DOI:** 10.1186/s12575-021-00142-y

**Published:** 2021-01-20

**Authors:** Wenjing Chen, Jing Ye Pan

**Affiliations:** 1 School of the First Clinical Medical Sciences, Wenzhou Medical University, Wenzhou, China; 2 Department of Intensive Care Unit, The First Affiliated Hospital of Wenzhou Medical University, Wenzhou, China; 3 Wenzhou Key Laboratory of Critical Care and Artificial Intelligence, Wenzhou, China

**Keywords:** SARS-CoV-2, COVID-19, Microcirculation Dysfunction, Autopsy, Pathology, Diffuse Alveolar Damage, Disseminated Intravascular Coagulation

## Abstract

The spread of the coronavirus (SARS-CoV-2, COVID-19 for short) has caused a large number of deaths around the world. We summarized the data reported in the past few months and emphasized that the main causes of death of COVID-19 patients are DAD (Diffuse Alveolar Damage) and DIC (Disseminated intravascular coagulation). Microthrombosis is a prominent clinical feature of COVID-19, and 91.3% of dead patients had microthrombosis.

Endothelial damage caused by SARS-CoV-2 cell invasion and subsequent host response disorders involving inflammation and coagulation pathways play a key role in the progression of severe COVID-19. Microvascular thrombosis may lead to microcirculation disorders and multiple organ failure lead to death.

The characteristic pathological changes of DAD include alveolar epithelial and vascular endothelial injury, increased alveolar membrane permeability, large numbers of neutrophil infiltration, alveolar hyaline membrane formation, and hypoxemia and respiratory distress as the main clinical manifestations. DAD leads to ARDS in COVID-19 patients. DIC is a syndrome characterized by the activation of systemic intravascular coagulation, which leads to extensive fibrin deposition in the blood. Its occurrence and development begin with the expression of tissue factor and interact with physiological anticoagulation pathways. The down-regulation of fibrin and the impaired fibrinolysis together lead to extensive fibrin deposition.

DIC is described as a decrease in the number of platelets and an increase in fibrin degradation products, such as D-dimer and low fibrinogen. The formation of microthrombus leads to the disturbance of microcirculation, which in turn leads to the death of the patient. However, the best prevention and treatment of COVID-19 microthrombosis is still uncertain.

This review discusses the latest findings of basic and clinical research on COVID-19-related microthrombosis, and then we proposed the theory of microcirculation perfusion bundle therapy to explore effective methods for preventing and treating COVID-19-related microthrombosis. Further research is urgently needed to clarify how SARS-CoV-2 infection causes thrombotic complications, and how it affects the course and severity of the disease. To cultivate a more comprehensive understanding of the underlying mechanism of this disease. Raise awareness of the importance of preventing and treating microthrombosis in patients with COVID-19.

## Introduction

COVID-19 is a new disease caused by severe acute respiratory syndrome. Coronavirus 2 (SARS-CoV-2) is spreading rapidly around the world. According to a report from the World Health Organization on January 8, 2021; more than 87 million cases have been confirmed worldwide, of which more than 1.8 million have died. It is not yet clear why the COVID-19 mortality rate has increased. Initially, the severe COVID-19 death was attributed to acute respiratory distress syndrome (ARDS), but recent coagulopathy and thromboembolic events are suspected to contribute to its high mortality [1]. The report revealed that in addition to the typical features of acute respiratory distress syndrome (ARDS), patients with COVID-19 also showed thrombotic necrosis of pulmonary capillaries [2]. Preliminary results of in vivo evaluation of sublingual microcirculation in patients with severe COVID-19 requiring mechanical ventilation indicate that thrombosis exists in the microcirculation. This finding strengthens that microvascular thrombosis is a sign of COVID-19. Another conclusion emphasized by the study is that microvascular thrombosis occurs systemically and may affect different organs. Those organs with high capillary density, such as the lungs, suffer the most. In the lungs, pulmonary microvascular thrombosis can lead to dead-end effects (ventilated areas, but not perfused), leading to the severe hypoxemia observed in these conditions. In turn, microvascular thrombosis may damage other areas, such as diffuse cortical necrosis and extensive thrombosis in renal biopsy, leading to extensive endothelial damage and microthrombosis [3, 4], liver and brain microthrombosis [5],leading to multiple organ dysfunction [6],autopsy series results show that the incidence of pulmonary artery capillary embolism is very high [7]. Human anatomy is the most direct method to reveal the true appearance of the virus, and it is one of the important methods for pathological research. Through the comparative study of pathological changes and clinical changes, reveal the pathogenesis, analyze the cause of death, summarize diagnosis and treatment experience, and improve clinical treatment and prevention effects.

In order to identify a new type of disease, an autopsy can provide us with many new answers. Since the first corpse of a COVID-19 deceased was conducted in China on February 16, 2020, there have been multiple pathological samples and autopsy reports, and related research work is also in progress.

We searched PubMed with “SARS autopsy”, “pathology of COVID-19”, “COVID-19 autopsy”, and “COVID-19 Microcirculation Dysfunction”, collected autopsy pathology reported during SARS and COVID-19, and explained pathological anatomy report and its application in clinical treatment. Chinese experts pointed out that in severe cases, patients can develop acute respiratory distress syndrome (ARDS), a disease with diffuse alveolar damage (DAD) as the main pathological manifestation.

Tang et al. [8] reported that 71.4% of COVID-19 patients who did not survive met the criteria for disseminated intravascular coagulation (DIC), while only 0.6% of survivors met the criteria. It is worth noting that the coagulation and fibrinolysis disorders in the pulmonary circulation and bronchoalveolar spaces may be important factors in the pathogenesis of ARDS in COVID-19.

Severe Acute Respiratory Syndrome Coronavirus 2 (SARS-CoV-2) enters cells through angiotensin-converting enzyme2 (ACE2) receptors to cause endothelial damage, which is believed to be the cause of diffuse endotheliitis.

Endothelial cell damage can lead to an inflammatory host response, which is characterized by excessive immune activation and cytokine storm, which promotes hypercoagulable state and thrombosis [9]. Therefore, this review will hopefully stimulate more interest and research, and it is important to raise awareness of the importance of microthrombosis in patients with COVID-19 and the importance of microthrombosis causing microcirculation disorders.

## Microscopic Pathology of SARS

1. Pulmonary lesions: extensive combination of both lungs; focal hemorrhage, necrosis, desquamative alveolitis and bronchitis, alveolar cavity is filled with proliferating alveolar epithelium and secreted proteins, monocytes, lymphocytes and plasma cells, transparent lung Membrane formation, changes in glomerular mechanized pneumonia in the alveolar cavity, mechanized exudation, and virus inclusion bodies can be seen in alveolar epithelial cells;
2. Immune organ damage: large necrosis of splenic lymphoid tissue, focal necrosis of lymph node, suppression of bone marrow hematopoietic tissue;
3. Systemic small vessel vasculitis: edema around small veins of the heart, lung, liver, kidney, adrenal gland, striated muscle and blood vessel wall, focal fibrin necrosis, infiltration of monocytes and lymphocytes, thrombosis in some small veins;
4. Changes in systemic toxicity: degeneration and necrosis of lung, liver, kidney, heart and adrenal parenchymal cells. Transmission electron microscopy of lung tissue showed aggregated virus particles [10].

## The General Pathology of SARS

1. Chest cavity and lungs: There is a small amount of pink or red liquid in the chest cavity, and no adhesions are seen in the pleura. The lung tissue is visible to the naked eye as dark red with tough texture.

It can still float on the water surface, and the light red or red liquid flows out from the longitudinal section, and there are few secretions in the trachea.

The upper left, lower left, upper right, middle right and lower right lobe were observed under multiple microscopes.

Except for a few fields, each lung lobe showed diffuse and persistent exudative lesions.

Alveolar capillaries are highly dilated and congested, and have some cavities.

Microthrombosis can be seen, due to edema, lymphocyte and monocyte infiltration, the alveolar wall is significantly thickened, and focal type II epithelial and mesenchymal cell active proliferation can be seen.

A light red uniform cellulose-like substance is deposited on the alveolar wall, the capillary basement membrane thickens, and the distance from the alveolar wall widens.

The alveolar epithelium is shed and incomplete.

Most of the alveolar cavity has red exudate.

It can be seen that there are cellulose, red blood cells, macrophages and epithelial cells in some alveolar cavities.

Virus inclusion bodies can be found in the alveolar epithelium. They are uniform red staining, structureless hollow bodies, and no multinucleated giant cells.

Bronchial submucosal edema, inflammatory cell infiltration, epithelial shedding or focal hyperplasia, more neutrophils and thromboembolism in the vessel lumen [11].

2. Hilar lymph node enlargement: The inherent structure of the lymph node disappears, the lymphatic sinus and blood vessels are obviously dilated, the number of lymphocytes in the cortical area is significantly reduced, and the germinal center is not seen. Macrophages proliferate in the subcystic lymphatic sinus. The liposome foam is swallowed. Visible focal necrosis [11]. Enlarged hilar and abdominal lymph nodes [12]
3. Spleen: The volume of the spleen decreases and the weight of the spleen decreases [12].

Under the microscope, white pulp atrophy, lymphocytes around the lymphatic sheath around the artery, sparse red pulp lymphocytes, and focal hemorrhage and necrosis can be seen in the lower part of the capsule [11].

4. Liver: The liver cells in the liver lobes are slightly swollen and fatty degeneration, the liver sinusoid cells are actively proliferating, the area of ​​the manifold is slightly enlarged, and a small amount of lymphocyte infiltration [11].
5. Heart: muscle fiber atrophy, brown pigmentation in the cells, mild proliferation of interstitial cells, and a small amount of lymphocyte infiltration [11].
6. Kidney: Cortical dysplasia, renal tubular edema, partially visible protein casts, glomerular fibrosis and corresponding renal tubular atrophy and fibrosis, glomerular concentration, and compensatory remnant glomerular hypertrophy [11]
7. Pancreas: mild acinar atrophy, a small amount of lymphocyte infiltration [11]
8. Small intestine: No obvious mucosal damage, submucosal hyperemia and edema, and other pathological changes under the naked eye and under the microscope, and a small amount of lymphocyte infiltration under the mucosa [11]

## The Relationship between SARS Pathology and Clinic

SARS virus infection is not limited to the lungs, but also involves other parts of the respiratory tract and other organ systems. The most important are immune cells, especially T lymphocytes, monocytes and macrophages [13]. Under antigen stimulation, NK and T cells rapidly secrete a large number of cytokines with immunomodulatory effects to enhance the body’s immunity against infection. SARS will invade the mucosa of the respiratory tract, lymph nodes and blood circulation after respiratory infection. SARS multiplies and multiplies, causing cell lysis and necrosis. Patients with reduced resistance are prone to mixed infections, and patients often die from complications of mixed infections. Almost every patient has diffuse serous inflammation in each lobe of the lung [14]. Focal necrosis of lung tissue, local multinucleated giant cells, and as the course of the disease prolongs, lung tissue fibrosis appears, this is a need for special attention Follow-up radiology studies on patient rehabilitation showed that 62% of the surviving patients had pulmonary fibrosis. Therefore, at least in severe SARS patients, pulmonary fibrosis may develop relatively quickly [15]. The patient’s gas exchange function was severely impaired, hypoxemia occurred, and acute respiratory failure, which is clinically called acute respiratory distress syndrome (ARDS). Finally, systemic arteritis and vasculitis lead to systemic microcirculation failure, and a large number of platelet aggregations show diffuse intravascular coagulation (DIC). If the patient can survive this dangerous period, the patient’s clinical symptoms will gradually improve. However, some patients, the mechanization of large alveoli, interstitial fibrosis and scar formation will damage the lungs and the gas exchange function of certain lung tissues will lose normal. Structure, lung compensatory capacity is reduced, lung capacity is reduced. The role of bronchioles and bronchiole in alveolar mechanization, interstitial fibrosis and scar formation. The lumen becomes thicker or thinner. Secretions accumulate in thickened areas, and thinning will affect the discharge of secretions. Basically, patients are susceptible to repeated infections caused by bronchial inflation. Pulmonary sclerosis also affects pulmonary circulation and right ventricular function, leading to pulmonary heart disease [16].SARS is a systemic disease that can damage many organs. The lungs, immune organs and small blood vessels throughout the body are the main targets of virus attack. Extensive consolidation of the lungs, diffuse alveolar injury, transparent membrane formation, respiratory distress and reduced immune function are the main causes of death [10]. Studies have found that SARS-CoV can infect vascular endothelial cells. This may indicate that direct infection of these cells is part of the observed endothelial damage. However, the lack of infected endothelial cells does not seem to explain the severe vascular damage [17]. Previous studies reported high levels of various cytokines and chemokines in the serum of SARS patients [18, 19]. Therefore, in addition to direct viral infection, the highly induced effects of pro-inflammatory mediators and hypoxia may play an important role in the vascular endothelial injury of SARS patients.

## Microscopic Pathology of COVID-19

1. Lungs: mainly the early exudation changes of the disease, pulmonary edema, alveolar fibrin exudation, inflammatory cells, alveolar epithelial cells, multinucleated meganuclei, hyaline membrane formation, and fibrin deposition. Type I alveolar cells in the alveolar cavity proliferated significantly, and some cells fell off and disappeared. Type II alveolar cells mainly proliferate and fall off. Inclusion bodies can be seen in type II alveolar cells and macrophages. Alveolar septal vascular congestion and edema, infiltration of monocytes and lymphocytes, and obvious thrombosis in the blood vessels. Hemorrhagic infarction may cause focal bleeding and lung tissue necrosis. Part of the alveolar exudate is mechanized and pulmonary interstitial fibrosis. The epithelium of the bronchial mucosa in the lung fell off, and the formation of mucus and mucus in the cavity was blocked. Rarely, the alveoli are over-inflated, the alveolar septum ruptures or forms cysts [20]. Histological examination revealed bilateral diffuse alveolar injury with cellular fibrous mucinous exudates. The right lung showed obvious lung cell shedding and hyaline membrane formation, showing acute respiratory distress syndrome (ARDS). The left lung tissue showed pulmonary edema and formed a hyaline membrane, suggesting early ARDS. Lymphocyte-based interstitial mononuclear inflammatory infiltration was found in both lungs. Multinucleated syncytial cells were identified in the alveolar cavity. Atypical pneumonia cells have larger nuclei, amphipathic granular cytoplasm, and have prominent nucleolus characteristics, showing viral cytopathy. No obvious nuclear or cytoplasmic virus inclusion bodies were found [21–23].

Coronavirus particles can be seen in the cytoplasm of bronchial mucosal epithelium and type II alveolar epithelial cells under electron microscope. Immunohistochemical staining showed that some alveolar epithelium and macrophages were positive for new coronavirus antigens, and RT-PCR detection of new coronavirus nucleic acid positive COVID-19 eosinophils had less encapsulation [21], SARS can be seen with more virus inclusions, and with transparent membranes and mucus plugs.

2. Spleen, hilar lymph nodes and bone marrow: The spleen is significantly reduced. In the spleen, the number of lymphocytes was significantly reduced, focal hemorrhage and necrosis, macrophage proliferation and phagocytosis were seen.

There are few lymphocytes in the lymph nodes and necrosis. Immunohistochemical staining showed that CD4 + T and CD8 + T cells in the spleen and lymph nodes were reduced, and the decline was more severe than SARS.

The third line of bone marrow cells decreased [20]. Lymph node tissue can be positive for the new coronavirus nucleic acid test, and the macrophage immunostaining for the new coronavirus antigen is positive.

Bone marrow hematopoietic cells may proliferate or decrease in number, and the proportion of red granules increases; hemophagocytosis is occasionally seen [20].

3. Liver and gallbladder: increase in volume, dark red. Hepatocyte degeneration, local necrosis with neutrophil infiltration; the gallbladder is highly filled [20]. Hepatic sinusoids are congested, lymphocytes and monocytes are infiltrated in the portal area, and microthrombosis is formed.
4. Heart and blood vessels: degeneration and necrosis of cardiomyocytes can be seen, and a small number of monocytes, lymphocytes and/or neutrophils infiltrate the matrix [20]. Clinically, markers of myocardial injury have increased significantly. In a small number of patients, 1/4 to 1/3 of the severe cases have increased myocardial enzymes, but there is no myocardial infarction. Pathological myocardial damage is not obvious. Endothelial cell shedding, intimal or full-thickness inflammation can be seen in small blood vessels in major parts of the body; mixed thrombosis, thromboembolism and infarction in corresponding parts can be seen in blood vessels. Visible thrombosis can be seen in the capillaries of the main organs [20].
5. Kidney: Impaired renal function; protein exudate can be seen in the glomerulus cavity, and the renal tubular epithelium degenerates and detaches. Interstitial hyperemia, microthrombosis and focal fibrosis can be seen. Protein exudate can be seen in the glomerular cavity, the renal tubular epithelium has degenerated and fallen off, and a transparent cast can be seen. Visible interstitial hyperemia, microthrombosis and focal fibrosis [20].
6. Urine and stool: Virus detected. Studies have shown that 4 out of 62 stool samples tested positive for the 2019 new coronavirus. Among the patients who tested positive on rectal swabs, four had detected the new coronavirus 2019 in the gastrointestinal tract, saliva or urine. In a case of severe peptic ulcer after the onset of symptoms, the 2019 new coronavirus was directly detected at the site of esophageal erosion and bleeding [24].
7. Other organs: brain tissue hyperemia, edema, degeneration of some neurons. The adrenal glands show focal necrosis. The mucosal epithelium of the esophagus, stomach and intestine is deformed and necrotic, falling off [20].

## The General Pathology of COVID-19

The lungs and pleura are moderately adherent, patchy and tough, with gray-white lesions and dark red bleeding. A large amount of viscous secretions overflowed from the surface of the alveoli, and fiber bundles were visible on the slices. The autopsy results were consistent with the distribution of imaging changes, and the lesions developed further. Considering the correspondence between the ground-glass shadows found in imaging and the gray-white alveolar lesions visible to the naked eye, it is considered that COVID-2019 mainly causes inflammatory reactions characterized by deep respiratory tract and alveolar damage [25]. There is not much pleural effusion, suggesting that there is no serous inflammation in lung disease [25]. Pericardial effusion, moderate epidural edema, and myocardial red flesh [25].

## The Relationship between COVID-19 Pathology and Clinic

COVID-19 shows early symptoms such as dry cough, foam-free sputum, and jelly-like substances in the alveoli. In terms of clinical features, the most common symptoms are fever (87.9%) and cough (67.7%), while diarrhea (3.7%) and vomiting (5%) are rare. 25.2% of patients have at least one underlying disease, such as hypertension, chronic obstructive pulmonary disease, etc. During hospitalization, the most common complication was pneumonia (79.1%), followed by acute respiratory disease (3.37%) and septic shock (1%) [24]. One of the distinguishing features of Covid-19 is that the lungs from the patient have extensive vascular thrombosis, accompanied by microvascular disease and alveolar capillary occlusion [26]. The main pathological manifestation of SARS infection is the formation of a hyaline membrane in the alveolar cavity, which eventually leads to consolidation of the lung, inability to breathe effectively at the site of the consolidation, and clinical respiratory distress. COVID-19 is different, mainly due to distal alveolar injury, mucus-like exudation, and inconspicuous lung hyaline membrane. From a clinical point of view, lymphocytes in COVID-19 patients are reduced, the pathological spleen is significantly reduced, the number of lymphocytes is significantly reduced, the body’s immune system is damaged, and the early disease is mainly exudative. Pulmonary inflammation, vasodilation, blood cells, white blood cells, fluid exudation, fibrinogen exudation into fibrin, entering the alveolar cavity that originally required gas exchange, and forming exudate are basic pathological changes. Later found interstitial fibrosis, mechanization and pulmonary complications. Pathology showed bronchial exudate, viscous secretions and inflammatory exudate mainly blocked the 14/15 grade bronchus. It is recommended to lie on the prone by tackling the sputum, slapping the back and changing the position of the body, which makes it easy to expel mucus. Although glucocorticoid therapy is not recommended for SARS-CoV-2 pneumonia routinely, according to the pathological results of pulmonary edema and the formation of pulmonary hyaline membrane, serious patients should consider timely and appropriate use of corticosteroids and ventilator support to prevent ARDS happened. Lymphopenia is a common feature of COVID-19 patients and may be a key factor related to disease severity and mortality [21]. Lung disease progresses rapidly and requires early respiratory treatment. Phlegm plugs are formed by mucous lesions in the lungs, which are pathological changes after disease damage. The formation of sputum plugs is not conducive to the effective delivery of oxygen from the ventilator to the alveoli, resulting in ineffective ventilation of the ventilator. Therefore, it is more effective to reduce sputum, suck sputum, clear the respiratory tract, and then provide oxygen support in clinical practice.

One of the focuses of clinical debate now is whether patients with new coronary arteries need early tracheotomy for invasive auxiliary respiratory support. Invasive tracheotomy is easier to remove the sputum in the main part of the deep respiratory tract, and it is easier to aspirate thick sputum. The problem of airway obstruction is easier to solve. However, airway incisions face a huge risk of respiratory aerosol infection. Therefore, more follow-up anatomical conclusions are needed to clarify whether the occurrence of mucus blockage in the patient’s airway is a common phenomenon, so as to determine which option should be clinically preferred.

## COVID-19 and Microthrombus

Some scholars proposed suggest the use of MicroCLOTS (microvascular COVID-19 lung vessels obstructive thromboinflammatory syndrome) as a new name for severe pulmonary coronavirus disease 2019 (COVID-19). They propose a mechanism of lung damage, primarily explained by a dramatic alveolar endothelial damage leading to a progressive endothelial pulmonary syndrome with microvascular thrombosis, and suggest MicroCLOTS (microvascular COVID-19 lung vessels obstructive thromboinflammatory syndrome) as an atypical ARDS working hypothesis [27].

A series of autopsy results showed that the incidence of microvascular thrombosis was high. Severe dysfunction of endothelial cells, massive thrombosis in the capillaries. Covid-19-related deaths are usually related to the immune system’s overreaction to viruses related to the so-called cytokine storm. It is recognized that the incidence of microthrombotic complications in COVID-19 patients has increased, and that microthrombosis is closely related to COVID-19. Autopsy of 22 patients who died of ARDS showed that 21 patients had thromboembolism. The large thrombus found at autopsy was related to the number of filling defects on angiography before death. Pulmonary microvascular thrombosis was once described as a complication of severe acute respiratory distress syndrome at autopsy, and later reports have been reported to complicate ARDS during outbreaks of other coronaviruses, including SARS-CoV and MERS-CoV. However, this feature is more pronounced in severe SARS-CoV-2 infections. Compared with influenza patients, the histology of COVID-19-related respiratory failure patients shows that the incidence of alveolar capillary microthrombosis has increased by 9 times [26] .One of the salient features of Covid-19 is that the patient’s lungs have extensive vascular thrombosis, accompanied by microvascular disease and alveolar capillary occlusion. In this regard, autopsy results show that, in addition to the expected features of diffuse alveolar injury found in ARDS, platelet fibrin thrombosis is a common microscopic finding in small pulmonary vasculature, and happened in 80–100% of lungs examined at autopsy Combined with other reported thrombotic events, the emerging microvascular thrombotic complications indicate that there is a strong interaction between COVID-19 and microcirculation disorders [28]. Here, we show that severe COVID-19 is characterized by the formation of highly neutrophil extracellular traps (NETs) in microvessels. The intravascular accumulation of NET leads to rapid blockage of the affected blood vessels, obstruction of microcirculation and organ damage. The accumulated NETs are related to endothelial injury, and a large number of microvascular congestion [1].

## Comparison of SARS and COVID

Common:

They are all RNA coronaviruses, their intermediate hosts are from wild animals, and their gene sequence homology is 79%.All CoV-S proteins bind to human airway mucosa angiotensin converting enzyme II (ACE2) and enter epithelial cells. The binding force of COVID-19 is 10–20 times higher than that of SARS-CoV, so it has stronger resistance to infection [22, 29].

Difference

a SARS has severely damaged many organs throughout the body, and the pathological changes of tissues and organs outside the lungs of COVID-19 are generally not obvious. The severity of the entire lung disease, especially the degree of alveolar damage and lung necrosis, is less severe than SARS. Unlike SARS, COVID-19 has slight damage to the alveolar wall, and SARS alveolar epithelium is severely shed [25].
b Both have type II alveolar epithelial hyperplasia. The proliferation of SARS decreased to the alveolar cavity, COVID-19 proliferated on the alveolar wall, and then the mass fell into the alveolar cavity. The degree is different.
c Degree and progression of alveolar fibrosis. After virus attack, epithelial cells and macrophages accumulate in the cytoplasm. After staining, the virus content is round pink [30], which is very common in SARS and difficult to find in COVID-19 [21]. This indicates that there are different forms of viruses, and the degree of aggregation of attacking cells is also different.
d Pulmonary fibrosis and comorbidities of COVID-19 are not as serious as SARS, and exudative lesions (mucus embolism) are more obvious than SARS. It may be related to the virus attacking mainly the serous glands (rather than the mucous glands). Serous gland cells are obviously damaged, and the mucous glands that are not fixed can secrete large amounts of mucus. A large amount of exudate is mixed with alveoli and bronchioles.
e COVID-19 extrapulmonary organs, splenic lymph node damage and flaky necrosis are slightly more severe than SARS. This indicates that the lung is a very important target organ, and the organs outside the lung, especially the immune system, are relatively damaged. This damage may be caused directly by the virus or indirectly caused by systemic cytokines. The SARS autopsy showed that the lungs showed severe diffuse alveolar damage (DAD) and bronchopneumonia, the spleen and lymph nodes showed lymphoid depletion, and the liver showed microvesicular steatosis.

The main pathological manifestation of SARS infection is the formation of a transparent membrane in the alveolar cavity, which eventually leads to consolidation of both lungs. The consolidation position cannot be effectively breathed, and clinical respiratory distress occurs. SARS The pathological changes of SARS can be summarized into four aspects: lung disease, immune organ damage, systemic small vasculitis, and systemic poisoning. Focal necrosis of the lung tissue, multinucleated giant cells can be seen locally, with the prolonged medical history, lung tissue fibrosis, this is a problem that deserves special attention after the patient recovers. COVID-19 is different. Distal alveolar injury is the main form, accompanied by mucus-like exudation, manifested by systemic vascular changes, pulmonary vasculitis, fibrinoid necrosis and thrombosis, and microthrombosis in the lungs- Diffuse thrombosis DIC.

## What Is the Incidence of Microthrombosis in COVID-19 Patients?

The number of autopsies collected this time was 334, and the number of microthrombi reached 305, accounting for 91.3%.

Published in the Lancet Medical Journal, autopsy of 10 African Americans whose cause of death was COVID-19 was performed. Important findings included thrombosis and microvascular disease in small pulmonary blood vessels and capillaries [31]. Autopsy of 3 patients who died of COVID-19 in Chongqing revealed that hyaluronic thrombus was found in a few capillaries [32]. Postmortem autopsy reports of 10 cases noted clear capillary thrombosis and mixed intravascular thrombus [33]. An autopsy report of 23 cases in Houston, Texas pointed out that acute COVID-19 pneumonia has distinctive features of acute interstitial pneumonia, diffuse alveolar injury components, microvascular involvement in intravascular and extravascular fibrin deposits, and neutrophils. It is trapped within the blood vessels and often forms microthrombi in the arterioles. Severe pulmonary thromboembolism with pulmonary infarction and/or hemorrhage occurred in 5 of 23 patients (21.7%) [34]. The autopsy results of 21 COVID-19 patients were reported. The main causes of death were respiratory failure, exudative diffuse alveolar injury and massive capillary congestion. Despite anticoagulation therapy, they were often accompanied by microthrombi. Five cases (23.8%) showed pulmonary thrombotic microangiopathy [35]. Autopsy of 12 COVID-19 patients showed microvascular thrombosis [36]. Autopsy of 67 COVID-19-positive patients was performed at Mount Sinai Hospital. In many patients, microthrombuses in multiple organ systems including the brain were reported. New findings reported include the endothelial phenotype of ACE2 in selected organs. This phenotype is related to coagulation abnormalities and thrombotic microangiopathies, and can solve prominent coagulopathy and neuropsychiatric symptoms. Another initial observation is that macrophage activation syndrome, with phagocytic and phagocytic lymphocytic histiocytosis, is the basis for microangiopathy and excessive cytokine release [37]. In the minimally invasive ultrasound-guided autopsy, 8 out of 10 (80%) patients had alveolar arteriole fibrosis thrombosis [38]. Some literature reveals that in addition to organ-specific changes, microscopic thrombosis, especially those found in the lung, kidney and prostate, is the most important observation under the microscope [39]. Autopsy of 3 cases of COVID-19 confirmed microthrombus [40]. The autopsy of 38 patients with COVID-19 revealed that in COVID-19, inflammatory microvascular thrombosis exists in the lung, kidney and heart [41]. In 7 patients, all autopsies showed platelet-rich thrombi in the microvessels of the lung, liver, kidney, and heart regardless of the anticoagulation status [5]. Eight autopsies of COVID-19 mentioned that COVID-19 may define a catastrophic microvascular injury syndrome mediated by complement pathway activation and related procoagulant states [42]. According to the autopsy of 14 cases of COVID-19 included in The Lancet, the main lung findings were acute or tissue-stage diffuse alveolar injury, and 5 cases (35.7%) of the patients showed focal pulmonary microthrombus [43]. An autopsy of 14 COVID-19 deceased was performed, and a key finding was the presence of thrombotic/thromboembolic vascular occlusion: capillary thrombosis (11/14, 78.6%) [44]. Autopsy of lung tissue samples of 38 patients with COVID-19 revealed 33 cases of platelet-fibrin thrombus (86.8%). The lung lesions of COVID-19 patients are mainly diffuse alveolar injury. Hyaline membrane formation and dysplasia of lung cells are common. Importantly, the presence of platelet-fibrin thrombi in arterioles is consistent with coagulopathy, which seems to be very common in COVID-19 patients and should be one of the main goals of treatment [45]. The report of the first German who died of COVID-19 suggests that a microthrombotic event occurred in the lungs [46]. The pulmonary manifestations of 4 cases of fatal COVID-19 are introduced. The early disease is characterized by microthrombosis and laboratory features of diffuse intravascular coagulation. Therefore, according to conventional standards, respiratory insufficiency may be considered unlikely to be the direct cause of death, but this situation and recently published autopsy data indicate that pulmonary microvascular changes are an important and distinctive feature of COVID-19, and may cause hypoxemia and acute cardiac insufficiency [47]. Standardized autopsy was performed on 13 patients who died of COVID-19. The autopsy revealed characteristic pathological changes in the lungs caused by COVID-19, which is believed to be the cause of death in most patients. The main histological finding was secondary alveolar injury, apparently due to focal capillary microthrombosis. Alveolar injury directly or by inducing pulmonary parenchymal fibrosis leads to death [48]. The autopsy results showed that the focal damage of microvascular pulmonary circulation is the main mechanism of fatal lung disease caused by SARS-CoV-2 virus. For patients recovering from severe COVID-19, this may also be the cause of persistent lung injury. Diffuse alveolar damage (DAD) was seen in 87% of the 68 autopsied lungs. The late incidence of DAD is relatively low and is related to the long duration of the disease. At least 84% of the lesions have fibrin microthrombi. In the ultrastructure, small blood vessels show duplication of basement membrane and obvious endothelial swelling, accompanied by cytoplasmic vacuolation. The report proposes that COVID-19 pneumonia is a heterogeneous disease (tracheobronchitis, DAD, and vascular injury), usually involving pulmonary blood vessels with capillary microthrombosis and inflammation and large thrombosis. Viral infection in the area of ​​persistent active injury leads to persistent and temporal heterogeneous lung injury [49]. Autopsy of 38 cases showed that organ involvement and the characteristics of thrombosis in COVID-19 were related to immune thrombosis. In COVID-19, inflammatory microvascular thrombi exist in the lungs, kidneys, and heart, including extraneutrophil traps associated with platelets and fibrin. COVID-19 patients also show neutrophil platelet aggregation and obvious neutrophil and platelet activation patterns in the blood, which vary with the severity of the disease. The abnormal immune thrombotic regulation of SARS-CoV-2 pneumonia is related to ARDS and systemic hypercoagulability [41]. In order to determine the role of microthrombosis in COVID-19, further work is needed.

## How Do we Construct Microthrombosis Associated with COVID-19?

The study found [45] that fibrin thrombosis (less than 1 mm in diameter) in arteriole vessels was observed in 87% of cases, and about half of the cases involved more than 25% of lung tissue and high levels of D-dimerization body. These findings may explain severe hypoxemia, which is a characteristic of ARDS in COVID-19 patients. Vascular microthrombus is often found in the area of ​​diffuse alveolar injury and is related to diffuse endothelial injury. Although these features are not pathogenic, they are common in our series, are widely distributed in the lung samples of the patients examined, and are the main unique vascular components. Although the underlying mechanism of COVID-19-related microthrombus has not been fully elucidated, it is conceivable that the main role of its development is the activation of the thrombotic inflammatory pathway induced by the “cytokine storm” caused by SARS-CoV2 infection. As with other forms of sepsis, endothelial dysfunction has been observed in COVID-19 patients leading to extensive microthrombosis, mainly located in the pulmonary vascular bed. In summary, these findings indicate that although the pathogenesis of COVID-19-related microthrombosis is multi-factorial, it is mainly located at the microvascular level, and the veins and arteries are involved in the later stage. This pathophysiological phenomenon raises some questions about the effectiveness of anticoagulants at full antithrombotic doses to effectively treat the disease [50].

Plasma D-dimer detection is a direct prognostic indicator of COVID-19. In this regard, D-dimer is a fibrin degradation product released when plasmin cleaves cross-linked fibrin. Compared with non-serious diseases, D-dimer levels appear to be higher in patients with severe COVID-19. Subsequently, further studies emphasized that the level of D-dimer in patients who did not survive COVID-19 increased, and that D-dimer continued to increase upon admission before dying [28].

## How Do we Deal with COVID-19 Microcirculation Disorder?

The Italian Thrombosis and Hemostasis Association provides some suggestions for the treatment of hemostatic disorders in COVID-19 patients based on expert consensus [51]. Including general management of patients. Strengthen the laboratory examination and monitoring of hemostasis and platelet count; it is recommended to use standardized procedures to collect clinical and laboratory data of all hospitalized patients to increase understanding of the disease; all hospitalized COVID-19 patients are strongly recommended to prevent venous thromboembolism; The use of a patient-based individual approach aims to balance the risk/benefit ratio of various anti-thrombotic strategies, while taking into account the potential hypercoagulable state; it is also recommended that all experts involved in the treatment of COVID-19 patients cooperate closely.

In fact, so far, there is no evidence that the SARS-CoV-2 virus exerts an inherent procoagulant effect. Therefore, the most reasonable hypothesis is that the virus activates the coagulation cascade by triggering a large-scale inflammatory response, similar to those observed in other forms of sepsis. Several studies have shown that thrombosis and inflammation, the two processes complement each other and closely interconnect each other [52]. That is, coagulation factors and platelets are directly involved in the regulation of the host’s immune response, showing its hemostatic effect independent of its pro-inflammatory function. In addition, the cytokine storm stimulates the expression of tissue factor on monocytes/macrophages and vascular endothelial cells, triggering a coagulation cascade on their surface. Thrombosis at the microvascular level leads to tissue ischemia and organ dysfunction [50]. Blood hypercoagulability and intravascular microthrombosis are the development nodes of severe COVID-19. Therefore, anticoagulation and anti-inflammatory therapy can be used as an important treatment strategy for severe COVID-19 [53]. Based on the successful experience of COVID-19 treatment in Wenzhou area, we propose the theory of microcirculation perfusion bundle [54].

## Microthrombosis: The Theory of Microcirculation Perfusion Bundle

So far there is no specific antiviral treatment method for SARS-CoV-2. Overall management considerations should include evaluation of concomitant infections in patients with critically ill sepsis, especially acute lung injury and ARDS. COVID-19-related coagulopathy should be managed like any other coagulopathy, including sepsis-related DIC. Patients who only suffer from COVID-19 coagulopathy may not develop SIC, DIC or have hemorrhagic diathesis or need blood component replacement. For those developing overt DIC, standard guidelines for blood composition support can be used. Microvascular thrombosis may also be the cause of multiple organ failure in patients with long-term infection, but the early lung dysfunction seems to be caused by inflammation, reaction and viral effects on lung tissue. Patients with sepsis should receive standard supportive care. Although the use of anticoagulants or other physiological drugs may reduce microvascular thrombosis and possible end-organ dysfunction, patients with sepsis or sepsis with SIC or open DIC should continue to receive preventive anticoagulation therapy. In addition, the D-dimer value of COVID-19 patients greater than 1 μg/mL is associated with fatal consequences. For these reasons, although their efficacy and safety are closely monitored, due to their anti-inflammatory properties, it has been suggested that anticoagulants may be beneficial in patients with severe COVID-19 [55–57]. Liquid anticoagulation mechanism, intravenous fluid infusion weakens the thrombin-fibrinogen interaction, coagulation factor XIII-fibrin polymer interaction, enhances the fibrinolysis reaction, and reduces platelet aggregation and adhesion. Hydration and anticoagulation are safe and effective.

## Discussion

Cytokine storm and endothelial dysfunction in patients with severe diseases, the disease progresses rapidly and is fatal. Endothelial dysfunction is the basic mechanism that triggers the procoagulant state, which eventually evolves into diffuse intravascular coagulation, leading to embolism of multiple organs, leading to multiple organ failure (MOF). The Italian Society of Clinical Hemorheology and Microcirculation aims to emphasize the role of microcirculation disorders in the pathogenesis of COVID-19, which is the biggest challenge facing the world health [58].

The general observation of anatomy is different from the previous local pathological features. In fact, this is the difference between puncture pathological biopsy and systemic pathological anatomy. Although the use of glucocorticoids is not a conventional treatment for COVID-19, the patient’s lung pathology is characterized by edema and transparent membrane formation, which indicates that timely and appropriate use of glucocorticoids and ventilator support in critically ill patients can help prevent. The development of ARDS. In addition, the signs of suffocation are not obvious, but according to the current understanding of deaths caused by new coronary pneumonia infection, this is largely related to breathing difficulties. The discovery of lung disease is currently the most clinically helpful and can help improve treatment.

However, there are still many individual differences in other evidence. It is recommended to follow-up pathological examination and follow-up pathological work. Infected patients are accompanied by basic diseases, and the pathological changes between old and new diseases affect each other. Different stages and different age groups need more pathological support. Currently, there is no specific antiviral treatment for SARS and COVID-19. Therefore, it is necessary to further study the infection mechanism of the coronavirus to determine a suitable therapeutic target.

## Conclusions

Among COVID-19 patients, especially those suffering from severe diseases and/or being sent to the intensive care unit, the incidence of microthrombotic complications has increased, and the coagulation system of most patients is activated. The presence of extensive microvascular thrombosis highly confirms the presence of microcirculation disorders. Extensive microthrombosis rather than pneumonia is the cause of respiratory failure. Autopsy studies found that pulmonary microvascular thrombosis may be the cause of severe hypoxia in patients with COVID-19.Smooth microcirculation can prolong the life of the patient; microcirculation disorders caused by microthrombosis and acute respiratory distress syndrome caused by diffuse alveolar damage are the main causes of death in COVID-19 patients.

## Data Availability

Not applicable.

## Abbreviations

DAD: Diffuse alveolar damage
DIC: Disseminated intravascular coagulation
ARDS: Acute respiratory distress syndrome
COVID-19: Coronavirus disease-19
SARS-CoV-2: Severe acute respiratory syndrome coronavirus 2
